# Distinct Roles for FOXP3^+^ and FOXP3^−^ CD4^+^ T Cells in Regulating Cellular Immunity to Uncomplicated and Severe *Plasmodium falciparum* Malaria

**DOI:** 10.1371/journal.ppat.1000364

**Published:** 2009-04-03

**Authors:** Michael Walther, David Jeffries, Olivia C. Finney, Madi Njie, Augustine Ebonyi, Susanne Deininger, Emma Lawrence, Alfred Ngwa-Amambua, Shamanthi Jayasooriya, Ian H. Cheeseman, Natalia Gomez-Escobar, Joseph Okebe, David J. Conway, Eleanor M. Riley

**Affiliations:** 1 Medical Research Council Laboratories, Fajara, Banjul, The Gambia; 2 Department of Infectious and Tropical Diseases, London School of Hygiene and Tropical Medicine, London, United Kingdom; 3 Institute of Medical Microbiology, Immunology and Parasitology, University Clinic Bonn, Bonn, Germany; Case Western Reserve University, United States of America

## Abstract

Failure to establish an appropriate balance between pro- and anti-inflammatory immune responses is believed to contribute to pathogenesis of severe malaria. To determine whether this balance is maintained by classical regulatory T cells (CD4^+^ FOXP3^+^ CD127^−/low^; Tregs) we compared cellular responses between Gambian children (n = 124) with severe *Plasmodium falciparum* malaria or uncomplicated malaria infections. Although no significant differences in Treg numbers or function were observed between the groups, Treg activity during acute disease was inversely correlated with malaria-specific memory responses detectable 28 days later. Thus, while Tregs may not regulate acute malarial inflammation, they may limit memory responses to levels that subsequently facilitate parasite clearance without causing immunopathology. Importantly, we identified a population of FOXP3^−^, CD45RO^+^ CD4^+^ T cells which coproduce IL-10 and IFN-γ. These cells are more prevalent in children with uncomplicated malaria than in those with severe disease, suggesting that they may be the regulators of acute malarial inflammation.

## Author Summary

While Tregs have been implicated in regulation of the immune response to chronic infections, their potential in determining disease outcome in acute infections is unclear. In this study we have found that Tregs are unable to control the florid inflammation during acute, severe *P. falciparum* malaria infections, suggesting that this component of the immunoregulatory arsenal may be rapidly overwhelmed by virulent infections. Further, we identified, for the first time in an acute human infection, a population of IL-10-producing Th-1 effector cells and found that IL-10-producing Th-1 cells were associated with development of uncomplicated as opposed to severe malaria, leading us to suggest that such “self-regulating” Th-1 cells may contribute to clearing malaria infections without inducing immune-mediated pathology. In addition, we found evidence that malaria-induced Tregs may limit the magnitude of malaria-specific memory responses detectable 28 days later, which may reduce the risk of immune-mediated pathology upon reinfection and may explain how immunity to severe disease can be gained after as little as one or two infections. We conclude that vaccines designed to induce cell-mediated responses should be assessed for their ability to induce IL-10 producing Th-1 cells and Tregs.

## Introduction

The clinical spectrum of *P. falciparum* infection ranges from asymptomatic parasite carriage to a febrile disease that may develop into a severe, life-threatening illness. The factors that determine disease severity are not completely understood but are likely to include both parasite and host components [1]–[3]. Ultimately, the interplay between the parasite and the immune response likely determines the outcome of the infection [4]. Although sterile immunity - completely preventing re-infection - is hardly ever seen and protection against clinical symptoms of uncomplicated disease is only acquired after repeated infections [5], immunity to severe disease and death may be acquired after as few as one or two infections [6] suggesting that different immune mechanisms underlie these different levels of immunity.

While there is a growing consensus that killing of malaria parasites or malaria-infected red blood cells requires the synergistic action of antibodies and cell-mediated immune responses [5],[7], the mechanisms conferring protection against severe disease are less clear. Given that pathology of severe disease has repeatedly been linked to sustained and/or excessive inflammatory responses [4], acquiring the ability to regulate these responses adequately may be a key determinant of immunity that protects against severe disease [8]. Thus, while an early inflammatory response is needed to control parasite replication in human *P. falciparum* malaria [9]–[11], excessive levels of pro-inflammatory cytokines such as TNF-α [12]–[15], IFN-γ, [16],[17], IL-1β and IL-6 [14],[18],[19] are associated with severe pathology. Conversely, low levels of regulatory cytokines such as TGF-ß have been associated with acute [20] and severe malaria [21],[22], a relative deficiency in IL-10 was seen in those who succumbed to severe malaria [23], significantly lower ratios of IL-10 to TNF-α were found in patients with severe malarial anaemia [24],[25], and high ratios of IFN-γ, TNF-α and IL-12 to TGF-β or IL-10 were associated with decreased risk of malaria but increased risk of clinical disease in those who became infected [26]. In summary, therefore, immunity against severe malaria may depend upon the host's ability to regulate the magnitude and timing of the cellular immune response, allowing the sequential induction of appropriate levels of inflammatory- and anti-inflammatory cytokines at key stages of the infection.

Given these associations between severe disease and exacerbated immune pathology, a number of studies have explored the role of CD4^+^CD25^hi^FOXP3^+^CD127^−/lo^ regulatory T cells (Tregs) in determining the outcome of malaria infection. Induced and/or activated in response to malaria infection [27], Tregs may be beneficial to the host in the later part of the infection - when parasitaemia is being cleared - by down-regulating the inflammatory response and thereby preventing immune-mediated pathology. On the other hand, if Tregs mediate their suppressive effects too early, this could hamper the responses required for initial control of parasitaemia, permitting unbridled parasite growth which may also lead to severe disease. Malaria-specific induction of Tregs has been observed in a variety of experimental malaria infections in mice [28]–[31], but their role in preventing severe malarial pathology is unclear. Thus, in BALB/c mice infected with a lethal strain of *P. yoelii*, ablation of Treg activity by depletion of CD25^+^ cells either allowed mice to control parasitaemia and survive [32] or had no impact on the course of disease [33]. Depleting CD25^+^ cells of BALB/c mice infected with either *P. berghei NK65* [34] or *P berghei ANKA* [35] reduced neither parasitaemia nor mortality, but increased the severity of symptoms in the diseased mice, suggesting at least some benefit from Tregs in this model. Rather oddly, infection of CD25^+^ T cell-depleted BALB/c mice with *P. chabaudi adami DS* led to increased parasitaemia and more severe anaemia [30]. Finally, CD25^+^ T cell depletion around the time of parasite inoculation reduced the incidence of experimental cerebral malaria in C57BL/6 mice infected with *P. berghei ANKA* in two independent studies [29],[31], but not when CD25+ T cells were depleted 30 days prior to infection [31]. Whilst various explanations have been offered for these discrepant results, including differences in the various strains of mice and parasites employed, the microbial microenvironment in which the mice are kept, and the precise CD25 depletion protocols employed, these studies are currently not very helpful when trying to understand the role of Tregs in human malaria infections.

Malaria naïve individuals undergoing experimental *P. falciparum* sporozoite infection showed an increase in *FOXP3* mRNA expression and expansion of Tregs 10 days after infection; Treg induction correlated with high circulating levels of TGF-β, low levels of pro-inflammatory cytokines and rapid parasite growth [27] suggesting - but not proving - that Treg activation early in infection may inhibit the development of effective cellular immunity. More recently, we have observed that Treg populations appear to be transiently expanded and activated during the malaria transmission season in individuals from a malaria endemic community [36], again suggesting that naturally acquired malaria infection can drive the expansion and activation of Tregs. However, although Tregs have been implicated in IL-10-mediated down-regulation of Th1-like responses in the placenta of malaria-infected women [37] and reduced Treg frequencies and function have been linked to enhanced anti-parasite immunity in certain ethnic groups in West Africa [38] the potential for Tregs to influence the clinical outcome of malaria infections is still unclear.

To investigate the role of Tregs during clinical malaria infection, we have compared cellular immune responses of children with either severe or uncomplicated malaria. Interestingly, although we did not observe any significant differences in Treg numbers or function between severe and uncomplicated malaria cases, our data do indicate that malaria-induced Tregs may limit the magnitude of malaria-specific Th1 memory responses and thus moderate pro-inflammatory responses to subsequent infections, providing a possible explanation for the very rapid acquisition of immunity to severe malaria. Moreover, we have identified a population of FOXP3^−^, CD45RO^+^, CD4^+^ T cells which co-produce IL-10 and IFN-γ and which are more prevalent in children with uncomplicated malaria than in those with severe disease. We suggest that these IL-10 producing effector T cells may contribute to clearing malaria infection without-inducing immune-mediated pathology.

## Results

Immune responses of 59 Gambian children with severe *P. falciparum* malaria were compared with those of 65 children with uncomplicated clinical malaria and with 20 healthy (control) children of similar age and recruited from the same study area at the same time (Table 1). On admission, only 12 (9.4%) patients had a white blood cell count (WBC) above the age-specific norm and there was no significant difference in median WBC count between uncomplicated and severe cases, suggesting that few if any children had a concomitant systemic bacterial infection. No difference was observed in the differential WBC between the two groups. As expected, [39]–[41] numbers of lymphocytes, CD3^+^ and CD4^+^ T cells were significantly lower during the acute disease than during convalescence in both the severe and the uncomplicated groups (Table 1A). Parasite density on admission was two-fold higher in patients with severe malaria than in those with uncomplicated malaria; severely ill children also had significantly lower hemoglobin levels and were on average 2.4 years younger than children with uncomplicated disease. The number of *P. falciparum* clones per clinical isolate ranged from 1 to 4 (with an overall mean of 2 (CI95%: 1.8–2.1)), and – as has been observed previously [42] - did not differ significantly between the three groups (p = 0.3, Table 1B). Other factors potentially confounding immune responses, such as the degree of malnutrition or intestinal helminth infections were of similarly low prevalence in both severe and uncomplicated malaria cases and were not associated with severity of disease (Table 1B).

**Table 1:** Characteristics of study participants (A) and distribution of potential confounding factors (B).

A)	Day 0		Day 28		p value	p value
	Severe	Uncomplicated	Severe	Uncomplicated	Severe vs. Uncompl	D0 vs. D28
WBC [×10e9/L]	9 (8.1–10.2)	7.3 (6.6–8.1)	7.5 (6.8–8.3)	6.6 (6.0–7.3)	0.199	0.116
Lymphocytes [×10e6/L]	2479 (2178–2821)	2286 (1926–2653)	3715 (3225–4230)	3289 (2826–3693)	0.638	<0.001
CD3+ PBMC [×10e6/L]	1761 (1439–2448)	1642 (1398–1922)	2998 (2291–3594)	2688 (2354–3034)	0.489	<0.001
CD4+ PBMC [×10e6/L]	958 (766–1378)	935 (798–1089)	1522 (1202–1859)	1389 (1216–1590)	0.721	<0.001
Neutrophils [×10e6/L]	5536 (4761–6232)	4418 (3781–5056)	3207 (2822–3635)	2745 (2480–3063)	0.131	<0.001
Monocytes [×10e6/L]	567 (494–652)	444 (393–504)	500 (445–558)	446 (398–496)	0.201	0.928
Platelets [×10e3/µl]	61 (48–91)	109 (96–128)	262 (221–301)	267 (248–290)	<0.001	<0.001
Hb [g/dl]	10.1 (9.45–10.6)	11.7 (11.3–12.1)	11.6 (11.2–12.1)	11.9 (11.6–12.3)	0.01	0.453
GM^a^ parasitaemia [/µl]	270,236 (210,693–346,606)	136,142 (96,924–191,228)			0.008	
Age [years]	4.4 (3.9–5.1)	6.7 (5.8–7.7)				
Weight [kg]	14.8 (13.5–16.5)	22 (19.3–25.4)				

B)	Day 0		Day 28		p value
	Severe	Uncomplicated	Severe	Uncomplicated	Severe vs. Uncompl
Number of participants [n]	59	65	48 (81.4%)	56 (86%)	
z score	−1.77 (−2.2 to −1.31)	−1.39 (−1.71 to −1.06)			0.19
Intestinal helminths	6.5% (1.8–20.7%)	5.5% (1.9–14.9%)			0.59
Sickle cell trait	5.3% (1.8–14.4%)	5.0% (1.7–13.7%)			0.636
Cough on presentation	27.1% (17.4–39.6%)	26.2% (17–38%)			0.532
Prior antimalarial treatment	34.5% (23.4–7.7%)	37.5% (26.7–49.7%)			0.444
Number of parasite clones	1.9 (1.65–2.07)	2.0 (1.81–2.27)			0.23

a Geometric mean.

Unless otherwise indicated, the median with a 95% CI is given for severe and uncomplicated cases for day 0 (left) and day 28 (right). P values for variables shown in part (A) are derived from the random effects linear regression model. Part (B) shows z-scores compared using Mann-Whitney test, and proportions for qualitative variables not included in the model, compared using Chi-square test.

For statistical analysis, patients were classified as uncomplicated or severe cases, with the latter being further subdivided into those patients suffering from cerebral malaria (CM), severe anaemia (SA) or severe respiratory distress (SRD) (grouped together as S_A_) and those suffering only from severe prostration (grouped as S_B_). Data were analysed using linear regression, with a random effect to allow for the within subject measurements over time, adjusting for age, sex, duration of prior symptoms and numbers of clones causing the infection. Due to the multiplicity of comparisons that were made within the model, resulting from multiple responses and multiple comparisons within response, hypotheses rejected with a probability of less than 0.012 have a false discovery rate of 5% [43].

### Similar numbers and proportions of Tregs among uncomplicated and severe malaria cases, increasing during convalescence

**Figure 1:**
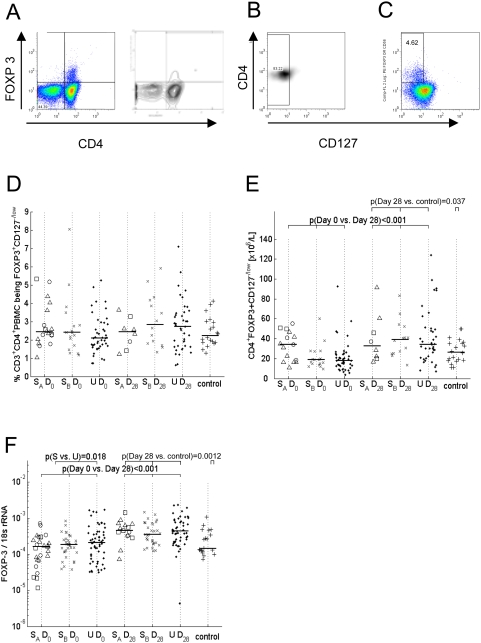
Similar number and proportion of Tregs in uncomplicated and severe disease, increasing during convalescence. Tregs were defined as CD3^+^CD4^+^PBMC being FOXP3^+^and CD127^−/low^. (A) CD3^+^ viable PBMC were displayed according to CD4 and FOXP3 and FOXP3 positive cells were defined on contour display as cells above the 90% contour level. (B) CD3^+^CD4^+^FOXP3^+^ PBMC were displayed according to CD127 expression to define the CD127 low cut off. (C) both gates were applied to CD3^+^CD4^+^ PBMC. (D) The percentage of Tregs for severe cases is shown for the subgroup (S_A_) comprising cases with CM (triangles), SA (squares) and SRD (circles), for the subgroup with severe prostration (S_B_), and for uncomplicated cases (U) for days 0 (D0) and 28 (D28) and compared to healthy controls of a similar age range recruited from the study area at the same time. (E) absolute numbers of cells expressing a Treg phenotype. (F) *FOXP3* mRNA levels expressed as a ratio to the house keeping gene *18s* rRNA. Dots represent individual data; bars represent the median. P-values are derived from the random effects linear regression model, adjusting for age, sex, duration of symptoms and numbers of clones isolated.

We hypothesized that children with severe malaria would have fewer circulating Tregs than children with uncomplicated malaria, or that Tregs of severely ill children would be less active than those of children with uncomplicated disease. However, the proportion of cells expressing a Treg phenotype (defined by flow cytometry as CD3^+^CD4^+^ lymphocytes being FOXP3^+^and CD127^−/low^; Figure 1A, 1B, and 1C) was similar in the acute (D0) and the convalescent phase (D28) for both uncomplicated and severe cases (S_A_+S_B_) and in healthy control children; on average, 2–3% of CD4^+^ T cells expressed the regulatory phenotype (Figure 1D). However, when the number of cells expressing a Treg phenotype was calculated using lymphocyte and monocyte counts from the differential WBC, we found that the absolute numbers of Tregs (per litre of blood) were significantly and similarly elevated in both severe and uncomplicated malaria cases during convalescence when compared to the acute phase (p<0.001) or when compared to the control group of healthy children (p = 0.037) (Figure 1E). A similar kinetic was observed for *FOXP3* mRNA levels (Figure 1F).

**Figure 2:**
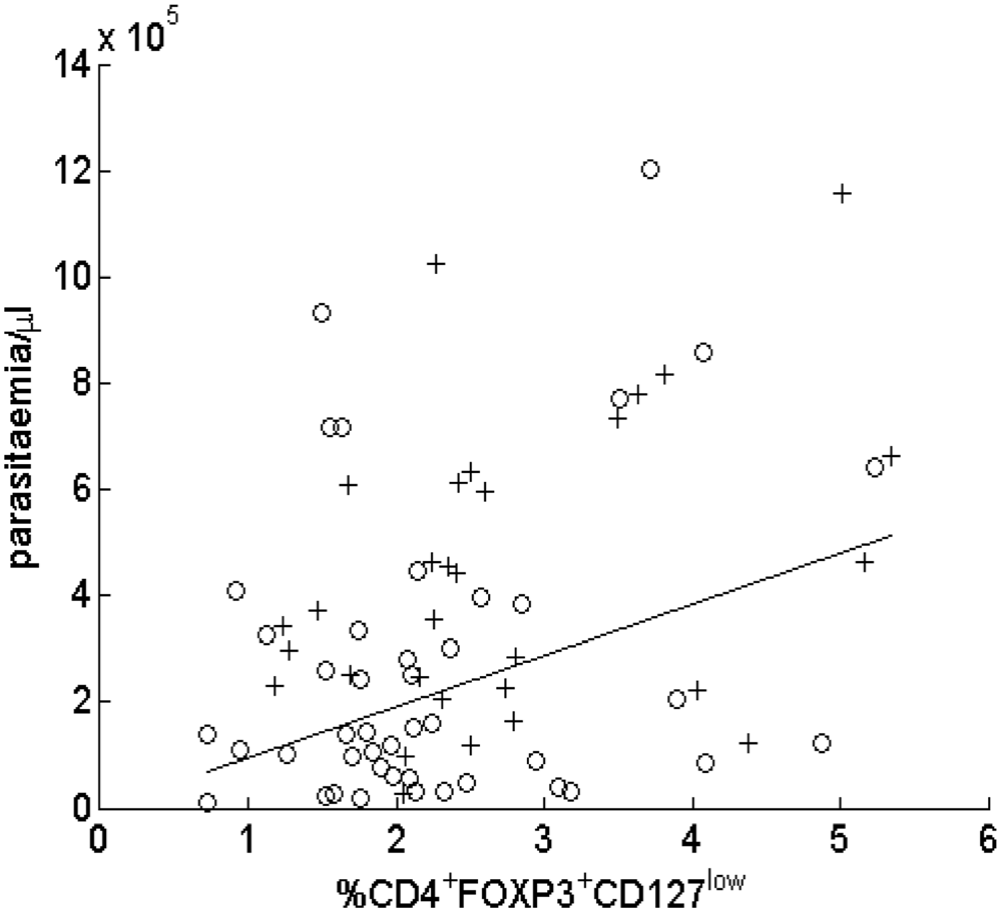
Proportion of T cells expressing a Treg phenotype correlates with parasitaemia. Parasite density in peripheral blood is plotted against the proportion of Tregs. +: severe cases; ○: uncomplicated cases. P value for linear regression ignoring disease status: 0.002. (gradient for separate lines for uncomplicated and severe cases not significantly different).

Although not supportive of our original hypothesis, these observations are consistent with the notion that acute malaria infection drives expansion of Treg populations which then persist for some weeks to maintain immune homeostasis during the contraction phase of the effector response [36]. In accordance with this notion, and in agreement with our previous observation that increased levels of Tregs were associated with faster parasite growth during the early stages of blood stage infection [27], we observe here in - children with either severe or uncomplicated malaria infections - a significant positive correlation between parasite density and the frequency of Tregs within the CD4^+^ T cell population (p = 0.002, Figure 2).

### Tregs display an activated memory phenotype during acute disease

**Figure 3:**
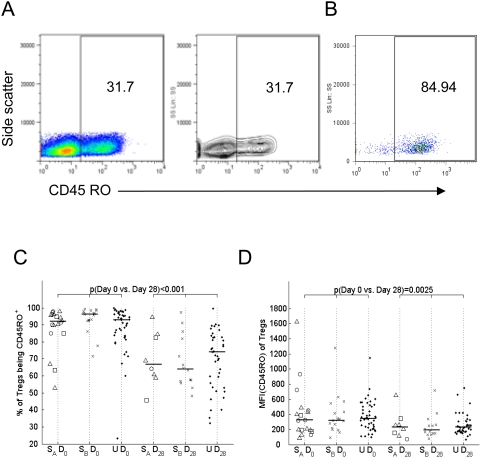
Tregs display an activated memory phenotype during acute disease. (A) Defining the gate for CD45RO staining on CD3^+^ PBMC using the contour display. (B) CD3^+^CD4^+^FOXP3^+^ CD127^−/low^ PBMC displayed according to CD45RO expression using the gate defined in (A). (C) percentage of CD3^+^CD4^+^FOXP3^+^ CD127^−/low^ PBMC expressing CD45RO. (D) Median Fluorescence Intensity (MFI) for CD45RO staining on CD3^+^CD4^+^FOXP3^+^ CD127^−/low^ PBMC. Symbols, bars and p-values as defined in Figure 1.

Since fully differentiated Tregs predominantly express an activated/memory phenotype [44] T cells from children with severe and uncomplicated malaria were analysed for expression of CD45RO (Figure 3A and 3B). In both uncomplicated and severe cases, the proportion of all T cells expressing CD45RO was significantly higher (p<0.001) during acute infection than during convalescence (data not shown). Irrespective of disease severity, more than 90% of Tregs expressed CD45RO during the acute phase of infection but expression of this marker decreased significantly (to approx 70%) during convalescence (Figure 3C). Likewise, the median fluorescence intensity (MFI) of CD45RO staining was 1.5 fold higher (p = 0.0025) during acute disease than during convalescence (Figure 3D). Taken together, these data indicate that in both uncomplicated and severe cases of malaria Tregs are predominantly of a memory phenotype and are activated during acute malaria infection.

### Similar Treg function in uncomplicated and severe malaria patients

**Figure 4:**
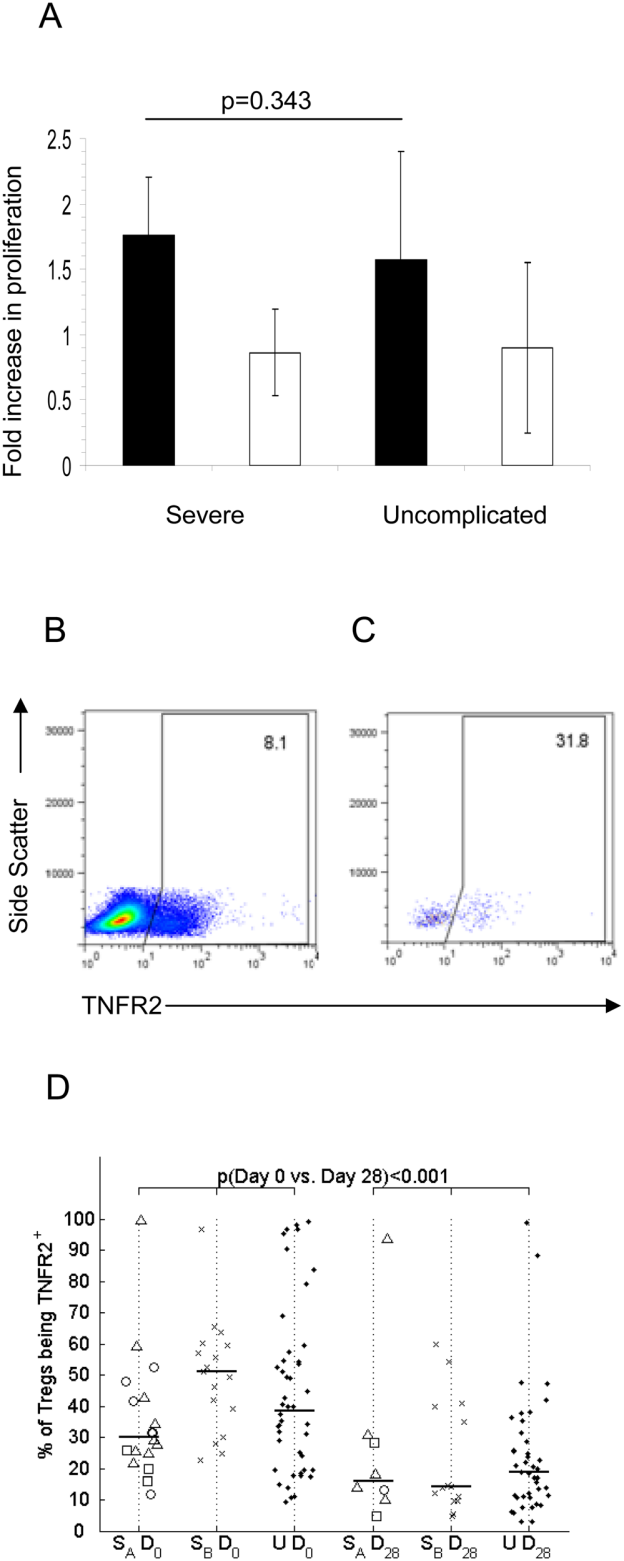
Tregs from severe and uncomplicated cases have similar functional capacity. (A) PBMC from 10 severe and 10 uncomplicated cases collected on day 0 were depleted of CD25+hi cells or mock depleted using magnetically labelled beads (Dynal), and assessed for proliferation using [3H]-thymidine after 6 days in culture with *P. falciparum* shizont extract (PfSE), uninfected red blood cells (uRBC), PMA+Ionomycin (P+I), or growth medium (GM), respectively. The geometric mean fold increase in proliferation with 95% CI is shown for the stimulation index of 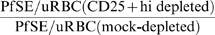 (black bars) or 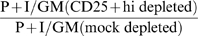 (white bars). (B) Representative examples of TNFR2 expression on PBMC, and (C) on Tregs. (D) Proportion of Tregs that express TNFR2. Symbols, bars and p-values as defined in Figure 1.

Three different indicators were used to assess the regulatory potential of Tregs during acute malaria infection. Firstly, using a classical anti-CD25 depletion assay, we assessed the ability of Tregs to suppress *P. falciparum* shizont extract (PfSE)-driven lymphocyte proliferation. Anti CD25 treatment removed approximately half (geometric mean 48.8%; CI95%: 41–58%) of the CD4^+^ T cells that were FOXP3^+^CD127^−/low^ and this was associated with a 1.76 fold and 1.57-fold (geometric means) increase in PfSE-induced lymphoproliferation in severe and uncomplicated cases, respectively, with no significant difference between the groups (p = 0.343, Figure 4A).

Next, since reduced expression of *SOCS-2*, a member of the suppressors of cytokine signaling family confined to Tregs [45], has been linked to impaired Treg function in Africans [38] we compared *SOCS-2* mRNA levels among severe and uncomplicated cases. While *SOCS-2* levels were found to be significantly reduced during acute disease compared to convalescence (p = 0.0076), no difference was observed between those with severe and those with uncomplicated malaria (data not shown).

Finally, since high concentrations of TNF-α have been reported to impair Treg activity (by upregulating and then signaling via TNFR2, leading to decreased *FOXP3* mRNA and protein expression [46]), and the functional impairment of Tregs observed in rheumatoid arthritis patients can be reversed by anti-TNF-α antibodies [47], we considered the hypothesis that the high levels of TNF-α seen in severe malaria patients [13],[15], might upregulate TNFR2 and impair Treg function. TNFR2 expression on Tregs was assessed by flow cytometry (Figure 4B and 4C). However, although a significantly higher proportion of Tregs expressed TNFR2 (Figure 4D) - with higher MFI (data not shown, p = 0.028) - during acute disease compared to convalescence, no difference was seen in TNFR2 expression on Tregs between severe and uncomplicated cases (Figure 4D). Moreover, there was no correlation between plasma levels of TNF-α and TNFR2 expression on Tregs, neither among severe or uncomplicated cases nor among all cases combined; neither did we observe any inverse correlation between TNF-α concentration and FOXP3 expression. Rather, the MFI of TNFR2 on Tregs was positively correlated with the MFI of FOXP3 in Tregs (r: 0.476; p<0.0001). Thus, our data seem to be more in line with data from mice suggesting that the interaction of TNF-α with TNFR2 on Tregs promotes their expansion and upregulation of FOXP3 [48] than with the data from studies of human rheumatoid arthritis.

### Stronger Th-1 responses observed in severe compared to uncomplicated cases are balanced by IL-10

**Figure 5:**
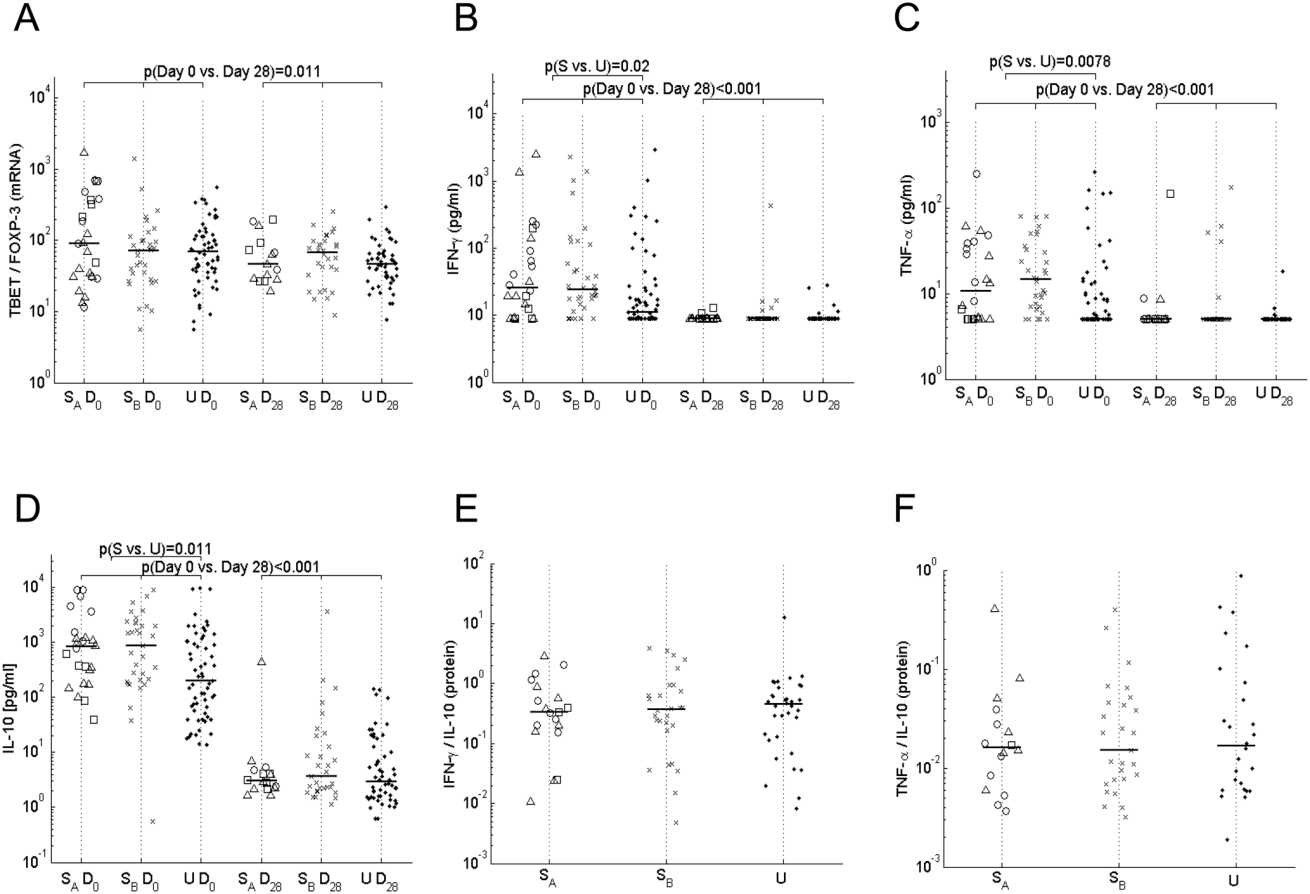
Ratios of Th1 over Treg cells and pro-inflammatory over regulatory cytokines. (A) Ratio of the mRNA level of the Th1 transcription factor *T-BET* over *FOXP3* for uncomplicated and severe cases during the acute phase (D0) and convalescence (D28) as determined by RT-PCR. Plasma levels for (B) IFN-γ, (C) TNF-α, and (D) IL-10 measured with a bioplex system and the ratios of (E) IFN-γ/IL-10 protein as well as (F) TNF-α/IL-10 protein on day 0. (NB: Protein ratios could not be calculated for Day 28, since cytokine concentrations were rarely above the detection limit of the assay. In Figure 5E and 5F only ratios for samples that had detectable responses for both cytokines on Day 0 are shown) Symbols, bars and p-values as defined in Figure 1.

Since the balance of T-effector to Treg responses is likely to be as important, or more important, than the absolute levels of either [26], we compared the ratio of the levels of mRNA for the Th1 transcription factor *T-BET* with those for *FOXP3*, currently considered the best marker for Tregs, and the ratio of T-effector cells (defined as CD3^+^CD4^+^CD25^+^FOXP3^−^, T-effector) over Tregs among the various groups. As shown in Figure 5A, in all groups the *T-BET*/*FOXP3* ratio was significantly higher during acute disease than during convalescence and a similar, albeit not significant, trend was observed for the ratio of T-effector/Tregs (data not shown). Moreover, since the absolute number of circulating T-effector cells was significantly higher in severe cases than in uncomplicated cases (p = 0.01, data not shown), the T-effector/Treg ratio tended to be higher among severe cases than uncomplicated cases on day 0 (p = 0.039) and a similar trend was seen for the *T-BET*/*FOXP3* ratio (p = 0.058). The ratio of *FOXP3* to *GATA-3* (Th2 lineage factor) mRNA was similar for both time points in all groups (data not shown) but the Th1/Th2 ratio (*T-BET*/*GATA-3* mRNA) was significantly higher during acute disease in children with CM, SA or SRD compared to those suffering from severe prostration (p = 0.0075), indicating that the expansion of the T-effector population is biased towards Th1 responses.

These data confirm previous studies indicating a shift towards a more inflammatory response during acute and severe malaria but, significantly, our data extend the previous observations by revealing that this inflammation is not balanced by a commensurate increase in Treg function. Indeed, our data strongly suggest that the potent inflammation induced during an acute malaria infection overwhelms the normal homeostatic capacity of the immune system and, in particular, that the Treg response in children with severe malaria is insufficient to balance a much stronger Th1 effector response.

To investigate further the dynamics of pro-inflammatory/regulatory responses during clinical malaria, plasma concentrations and mRNA transcripts of inflammatory (IFN-γ, TNF-α) and regulatory cytokines (IL-10) were assayed. In accordance with previous observations, plasma concentrations of IFN-γ, TNF-α and IL-10 were all significantly higher during acute disease than during convalescence, with significantly higher levels in severely ill children compared to uncomplicated cases (Figure 5B, 5C, and 5D). Levels of mRNA transcripts for *IL-10* and *IFN-γ* were also significantly elevated in all groups during acute disease, but there was no significant difference between severity groups (Figure S1A and S1B). For both severe and uncomplicated cases, levels of *IFN-γ* mRNA were highly correlated with levels of *IL-10* mRNA during the acute phase (severe: r = 0.833 p<0.001, uncomplicated: r = 0.693 p<0.001), suggesting that IFN-γ production is being balanced by IL-10 production. Interestingly, *IFN-γ* mRNA levels on day 0 correlated with *FOXP3* mRNA on day 0 for both severe (r: 0.39 p = 0.003) and uncomplicated cases (r: 0.44 p = 0.0001), suggesting that *IFN-γ* may also be driving *FOXP3* expression.

The balance of pro-and anti inflammatory cytokine responses clearly changed with time, but somewhat surprisingly, there were no marked differences in cytokines ratios between children with differing levels of disease severity. Thus, the ratios of TNF-α or IFN-γ to IL-10 on day 0 were similar in all disease severity groups (Figure 5E and 5F) and ratios of *IFN-γ* to *IL-10* mRNA were similar in all disease severity groups both on day 0 and day 28 (Figure S1F). However, *IFN-γ* mRNA levels were on average only 3.2-fold higher on day 0 than day 28 but *IL-10* mRNA levels were 29-fold higher on day 0, resulting in a significantly lower *IFN-γ*/*IL-10* mRNA ratio on day 0 than day 28 (Figure S1F).

### CD25^−^FOXP3^−^ CD45RO^+^ T cells but not Tregs are the major source of IL-10 during acute malaria

**Figure 6:**
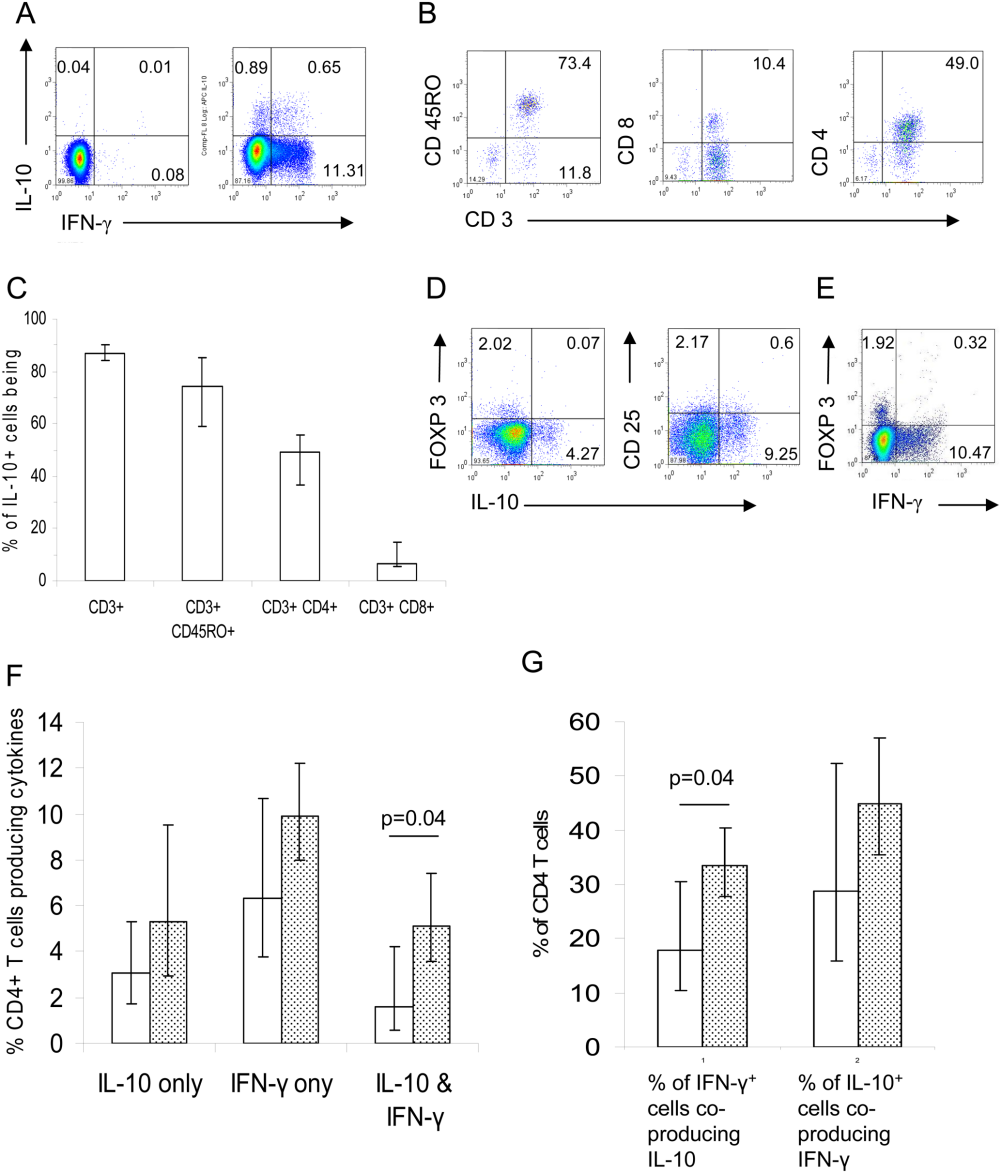
Phenotypic characterization of cytokine-producing cells. PBMC from 30 children with acute *P. falciparum* infection were cultured with growth medium (GM) or pulsed with PMA and Ionomycin (P/I) for 5 hours and assessed for the production of IL-10 and IFN-γ by flowcytometry. (A) representative example of PBMC stained with isotype control (left panel) or anti- IL-10 and anti-IFN-γ (right panel). (B) Representative example of the phenotype of IL-10 producing cells. (C) Proportion of IL-10 producing cells. The geometric mean with CI95% is shown. (D) Representative example of co-staining for IL-10 and FOXP3 (left panel) or CD25 (right panel). Across all samples tested, 0.13% (median; CI 95%: 0.09–0.16%) of PBMC stained positively for both IL-10 and FOXP3, corresponding to 2.4% (median; CI95%: 1.7–4%) of IL-10 producing cells being FOXP3^+^. Approx 0.79% (median; CI95%: 0.69–1.1%) of CD3^+^CD4^+^ PBMC were IL-10^+^ and CD25^+^, corresponding to a 8.68% (median; CI 95%: 4.9–9.8%) of IL-10^+^T cells being CD25^+^. (E) Representative example of co-staining for IFN-γ and FOXP3. Across all samples tested, 0.29% (median; CI 95%: 0.21–0.37%) of PBMC stained positively for both IFN-γ and FOXP3, corresponding to 0.97% (median; CI95%: 0.67–1.27%) of IFN-γ producing cells being FOXP3^+^. (F) Percentage of IL-10 single positive, IFN-γ single positive, and IL-10/IFN-γ double positive CD4^+^ T cells from 17 children with severe (open bars) and 13 children with uncomplicated (shaded bars) malaria are shown. PBMC were activated with P/I for 5 hours prior to staining. (G) The percentage of IFN-γ producing CD4^+^ T cells co-producing IL-10, as well as the percentage of IL-10 producing CD4^+^ T cells co-producing IFN-γ is shown for severe (open bars) and uncomplicated (shaded bars) cases. Bars in (F) and (G) represent the geometric mean with 95% CI.

IL-10 is a crucial immunoregulatory cytokine in both human [23] and murine [33],[49] malaria; we have recently identified CD4^+^ T effector cells as a major source of IL-10 [33], but the source of IL-10 in human malaria infection is unknown. In other protozoal infections of mice CD4^+^ effector T cells that co-produce IFN-γ and IL-10 have been identified [50]–[52]. We therefore cultured freshly isolated PBMCs from 30 children with acute malaria (17 severe, 13 uncomplicated) and 20 healthy control children, with or without PMA and Ionomycin (PI), for 5 hours and analyzed them for the presence of intracellular IL-10 and IFN-γ by flow cytometry (Figure 6A). No cytokine production was observed in unstimulated cells (data not shown), and PBMC from healthy children failed to produce any IL-10 in response to PI (data not shown), indicating that stimulation with PI predominantly induces cytokine production from recently activated cells.

By contrast, distinct populations of IL-10^+^ and IFN-γ^+^ cells were seen among the PI-stimulated cells from children with acute severe or uncomplicated malaria, with a small but easily distinguishable population of cells (approx 1% of all PBMC) producing both cytokines simultaneously (Figure 6A, right plot). In both severe and uncomplicated cases, IL-10 producing cells were predominantly CD45RO^+^ CD4^+^ T cells (Figure 6B and 6C) and were almost exclusively FOXP3^−^ and CD25^−^ (Figure 6D). Moreover, although a transient increase of FOXP3 in activated human T-effector cells has been reported [53], in our hands less than 1% (median 0.97%, CI95%: 0.67–1.27%) of IFN-γ producing cells were FOXP3^+^ (Figure 6E).

Overall, among children with acute malaria, approx 4% of PI-stimulated CD4^+^ T cells produced IL-10 and approx 8% produced IFN-γ and neither the proportions of cells producing one or the other cytokine (Figure 6F) nor the ratio of IFN-γ/IL-10 producing cells (data not shown) differed significantly between severe and uncomplicated cases. However, intriguingly, the proportion of CD4^+^ T cells simultaneously producing IL-10 and IFN-γ was three fold higher in uncomplicated cases than severe cases (geometric mean 5.2% vs 1.6%, p = 0.041, Figure 6F). Moreover, the proportion of IFN-γ^+^ CD4^+^ T cells that also produce IL-10 was almost twice as high among uncomplicated cases as among severe cases (p = 0.045, Figure 6G).

Taken together, these data indicate that during acute, uncomplicated or severe, malaria infections IL-10 producing cells are overwhelmingly T effector cells and that Th1 effector cells that also produce IL-10 are more prevalent in children with uncomplicated malaria than in children with severe malaria.

### The frequency of Tregs during acute disease is negatively associated with the magnitude of subsequent malaria-specific IFN-γ memory responses

It has been reported that Tregs present at the time of infection [54] or vaccination [55] may restrict the development of subsequent Th1 memory responses. To determine whether Tregs present during acute malaria infection might similarly affect the induction of immunological memory, we compared *FOXP3* mRNA levels on day 0 with malaria specific IFN-γ memory responses (as assessed by PfSE-specific cultured ELISPOT) among PBMC collected from 34 of our convalescent malaria patients (19 severe and 15 uncomplicated) on Day 28.

**Figure 7:**
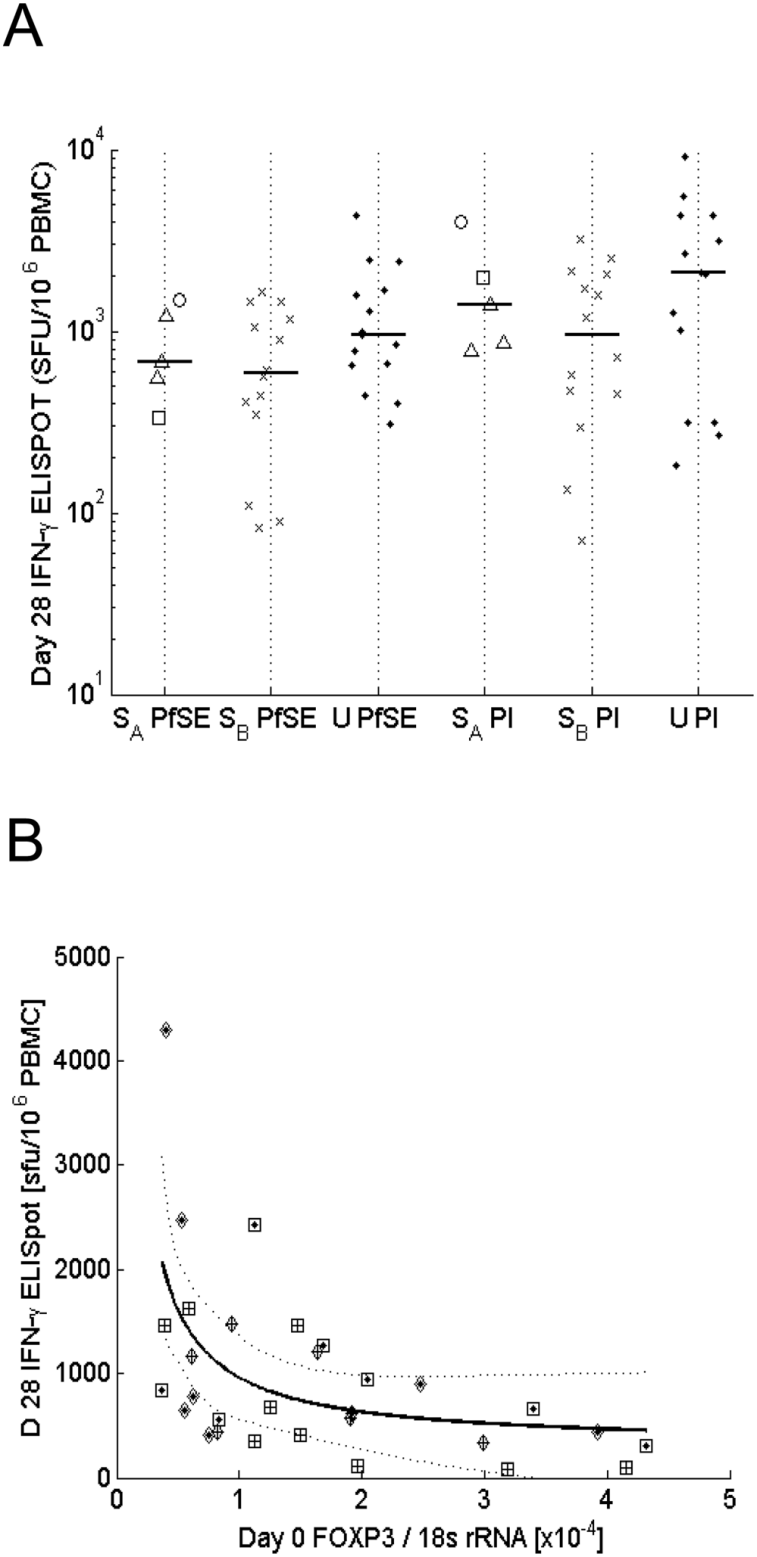
Tregs induced during the clinical episode under study limit the magnitude of subsequent malaria-specific IFN-γ memory responses. (A) PBMCs collected on day 28 were cultured for 6 days with PfSE, uRBC, P+I or GM, respectively, and assayed in an IFN-γ ELISpot to measure the magnitude of the malaria specific memory response. Background levels of IFN-γ production obtained in uRBC or GM were deducted from PfSE or PI. (B) cultured IFN-γ ELISpot responses to PfSE (D 28) are plotted against *FOXP3* mRNA levels measured on day 0. A linear by linear hyperbolic fit revealed a significant association (p = 0.009). +: severe cases; ○: uncomplicated cases. Scattered lines indicate the 95% CI limits. At the 5% level there is no significant improvement in fit, using separate lines for severe and uncomplicated patients. No significant effect of parasitaemia could be observed. Depending on a parasitaemia level above or below the median parasitaemia in both groups, a diamond (high) or a square (low) was added to individual data points to illustrate this.

As shown in Figure 7A, cells from uncomplicated and severe cases mounted similarly strong IFN-γ memory responses following culture with PfSE. When plotted against *FOXP3* mRNA levels measured on day 0, a linear by linear hyperbolic fit revealed that higher levels of *FOXP3* mRNA on day 0 were highly significantly (p = 0.009) associated with lower malaria-specific IFN-γ memory responses on Day 28, suggesting that Tregs induced during the acute infection may limit the magnitude of subsequent Th1 responses (Figure 7B). For neither group could a significant effect of parasitaemia on the memory response be observed (r = −0.12 p = 0.962 for severe and r = 0.015, p = 0.957 for uncomplicated cases).

## Discussion

We hypothesized that the balance of inflammatory to regulatory immune responses would be biased towards a more inflammatory response in children with severe malaria than in children with uncomplicated malaria, that this balance would be restored during convalescence and – crucially – that this would be associated with differences in the proportion, absolute number or function of circulating classical (CD4^+^ CD127^−/lo^ FOXP3^+^) regulatory T cells. In partial support of these hypotheses, the number of cells expressing a Treg phenotype and *FOXP3*-mRNA levels were both significantly higher during convalescence than during the acute clinical episode and the ratio of the Th1 transcription factor *T-BET* to the Treg transcription factor *FOXP3* was significantly higher during acute disease than during convalescence in both severe and uncomplicated cases, compatible with the notion that Tregs fail to sufficiently regulate pro-inflammatory responses which might contribute to the onset of symptomatic malaria infection.

Given our previous observation of Treg expansion during the pre-patent phase of malaria infection [27], we suggest that Tregs are induced/activated shortly after parasite emergence from the liver, that their numbers in peripheral blood then decline as a result of sequestration of CD4^+^ T cells during acute disease [40],[41],[56] and then, as has been described for other T cell subsets [39],[57], Tregs regain access to the circulation after malaria is cured. The significant positive correlation of Treg numbers with parasitaemia, as well as the correlation between *FOXP3* mRNA and *IFN-γ* mRNA levels in acute samples, further supports the notion that the initial infection induces a proportional increase in Tregs, attempting to balance the effector T cell response, and is in line with the recently proposed concept that antigenic challenge will give rise to an antigen specific Treg response, proportional in size to the inflammatory response [58]. Moreover, the Tregs circulating during acute malaria infections almost exclusively expressed an activated memory phenotype suggesting that they have expanded from a pre-existing pool of memory T-cells. This interpretation would be in line with recent elegant work in humans demonstrating that Tregs are derived by rapid turnover of memory populations in vivo [59], and with data from murine studies where, after CD25-depletion, malaria infection very rapidly drives differentiation of Tregs from circulating mature CD4^+^ T cells [60]. Obviously, it would be of interest to study the relationship between Tregs and effector T cell kinetics and parasite biomass, which is not readily measurable. Future studies may explore the usefulness of *P. falciparum* Histidine Rich Protein 2 in this context, which has recently been suggested as a surrogate marker for parasite biomass [61].

However, despite clear evidence of Treg induction and reallocation during acute malaria infection, we could not find any robust differences in Treg parameters between children with severe and uncomplicated disease. Thus, neither Treg numbers nor *FOXP3* mRNA levels differed significantly between children with uncomplicated malaria and those with severe malaria, and three different indicators of Treg function - their capacity to suppress lymphoproliferation, their expression of *SOCS-2* [45] and TNFR2 [46],[48] were all similar in severely ill children and children with uncomplicated disease. Furthermore, the similar distortion in the *T-BET*/*FOXP3* mRNA ratio during acute disease and the lack of any marked differences between the two groups in ratios of inflammatory to anti-inflammatory cytokines, as well as the close correlation between IFN-γ and IL-10 in both groups which is in line with previous observations in experimental human malaria infections [62], suggests that the systemic shift towards a pro-inflammatory immune response is similar in children with either severe or uncomplicated disease.

At first glance, these data do not appear to support the hypothesis that deficiencies in Treg function underlie the tendency of some children to develop severe, life threatening malaria.

However, we did observe significantly higher Th1 effector responses (more T-effector cells, higher concentrations of IFN-γ and TNF-α) in severely ill children than in children with uncomplicated disease, suggesting that the classical FOXP3^+^ Treg response that develops during acute malaria infection may be insufficient to balance the florid effector T cell response that develops particularly in children with severe disease. This would be in line with evidence showing that as the strength of the inflammatory stimulus increases, the suppressive capacity of human Tregs declines and the resistance of T-effector cells to regulation increases [63]. The situation observed during an acute, clinical malaria infection is thus in clear contrast to the situation in healthy, malaria-exposed individuals where Treg numbers closely track numbers of T-effectors, precisely maintaining an apparently optimal T-effector∶Treg ratio [36].

IL-10 is well-established as a vital homeostatic regulator of malaria-induced inflammation that prevents immune-pathology in mice [49],[64], promotes the necessary switch from early Th1 to subsequent Th2 responses [65],[66], and has been linked to protection from severe malaria anaemia [24],[25], and death [23] in humans. However, the cellular source of IL-10 in human malaria cases was, until now, ill defined. Contrary to our expectations, but in striking agreement with observations in *P. yoelii*-infected mice [33], CD45RO^+^ CD4^+^ T cells (that are CD25^−^ and FOXP3^−^) and not classical Tregs are the only substantial source of IL-10 during acute malaria infection. This observation is reminiscent of that made by Nylen [67] in patients with acute visceral leishmaniasis. Moreover, in our patients, a significant proportion of IL-10 producing CD4^+^ T cells were simultaneously producing IFN-γ, identifying them as Th1 cells. Although IL-10 secreting Th1 cells have been described recently in two murine models of toxoplasmosis [50], and cutaneous leishmaniasis [51], as far as we are aware, this is the first demonstration of IL-10 producing Th1 cells during human infections. Intriguingly, the proportion of these cells within the total CD4^+^ T cell population was significantly higher in children with uncomplicated malaria than in children with severe malaria suggesting that in human *P. falciparum* infection, as in murine *T.gondii* infections [50], IL-10 producing Th1 cells, activated by a strong inflammatory stimulus, may act as anti-parasitic effector cells with a “built in” control mechanism to prevent the onset of immune pathology. If so, then the ability of these self-regulating effector cells to localize to sites of parasite sequestration in tissues, where they mediate parasite killing whilst simultaneously blocking tissue damage, may be key to clinical immunity to malaria. Thus, our data strongly suggest that the percentage of IL-10-producing Th1 effector cells, rather than the cocktail of circulating cytokines, may be the most relevant biomarker of effective immunity to severe malaria.

Although Tregs may not seem to determine the outcome of current *P. falciparum* infections we did find evidence that they affect the magnitude of the malaria specific memory response induced by the current infection. A similar observation has been made in *P. berghei ANKA*-infected mice; animals that were depleted of CD25^+^ cells prior to infection and drug-cured on day 5 developed significantly stronger IFN-γ memory responses on day 14 than did intact infected/cured mice, and these mice also developed much more severe, and frequently fatal, clinical symptoms upon reinfection, despite more efficient parasite clearance [35]. Thus, malaria specific Tregs acquired during a primary infection may limit the magnitude of Th1 effector responses to subsequent infections to a level that allows parasite clearance without causing immunopathology. Future studies should be designed to test the hypothesis that Tregs may contribute to the very rapid development of resistance to severe malaria.

In summary, our data indicate that classical FOXP3^+^ Tregs are unable to control the florid inflammation that accompanies acute malaria infections and this component of the immunoregulatory arsenal is rapidly overwhelmed in children with either mild or severe malaria. Importantly however we have identified, for the first time in an acute human infection, a population of IL-10 producing Th1 effector cells which appear to be a major source of this key anti-inflammatory cytokine during acute malaria infection, and which are associated with development of uncomplicated as opposed to severe malaria. We propose that IL-10-producing Th1 cells may be the essential regulators of acute infection-induced inflammation and that such “self-regulating” Th1 cells may be essential for the infection to be cleared without inducing immune-mediated pathology. Moreover, we have found evidence in support of the hypothesis that Tregs limit the magnitude of the Th1 memory response raising the intriguing possibility that they may play an important role in the rapid evolution of clinical immunity to severe malaria.

## Materials and Methods

### Subject recruitment, study design, and study procedures

A case-control study was conducted in Gambian children with severe or uncomplicated malaria, resident in a peri-urban area within a 40 km radius south of the capital, Banjul, with low levels of malaria transmission [68],[69]. Patients were enrolled at Brikama Health Centre, the MRC Fajara Gate Clinic or the Jammeh Foundation for Peace Hospital in Serekunda between September 2007 and January 2008, after written informed consent was obtained from the parents or guardians. Uncomplicated disease was defined as an episode of fever (temperature >37.5°C) within the last 48 hours with more than 5000 parasites/µl detected by slide microscopy. Severe disease was defined using modified WHO criteria [70]: SA, defined as Hb<6 g/dl; SRD defined as serum lactate >7 mmol/L; CM defined as a Blantyre coma score ≤2 in the absence of hypoglycaemia, with the coma lasting at least for 2 hours. To avoid the confounding effects of other pathogens in children with concomitant systemic bacterial infections [71], children with clinical and/or laboratory evidence of infections other than malaria were not enrolled into the study. For some experiments, healthy children of the same age and recruited from the same area at the same time of the year were enrolled as controls. In total, 59 severe, 65 uncomplicated and 20 control cases were enrolled.

On admission (D0) and after 4 weeks (D28±3 days) one ml of blood was collected in RNA stabilizing agent (PAXgene™ Blood RNA system, Pre-AnalytiX) and a maximum of 4 mls of blood (mean: 3.2 mls CI 95%: 3.1–3.3 mls) were collected into heparinized vacutainers® (BD). All patients received standard care according to the Gambian Government Treatment Guidelines, provided by the health centre staff. The children's health was reviewed 7 days after admission. The study was reviewed and approved by the Joint Gambian Government/MRC Ethics Committee and the Ethics Committee of the London School of Hygiene & Tropical Medicine (London, UK).

*P. falciparum* parasites were identified by slide microscopy of 50 high power fields of a thick film. Full differential blood counts were obtained on days 0 and 28 using a Medonic™ instrument (Clinical Diagnostics Solutions, Inc); the presence of intestinal helminths was assessed by microscopy from stool samples collected into BioSepar ParasiTrap® diagnosis system, following the manufacturers' instructions. Sickle cell status was determined by metabisulfite test and confirmed on cellulose acetate electrophoresis [72].

### Cell preparation

Blood samples were processed within 2 hours of collection. Plasma was removed, stored at −80°C and replaced by an equal volume of RPMI 1640 (Sigma-Aldrich). PBMC were isolated after density centrifugation over a 1.077 Nycoprep (Nycomed, Sweden) gradient (800 g, 30 min) and washed twice in RPM 1640. Cells were either stained for flow cytometry directly *ex-vivo*, or cultured in RPMI 1640 containing 10% human AB+ serum, 100 µg/ml streptomycin, 100 U/ml penicillin (all Sigma-Aldrich), and 2 mM L-glutamine (Invitrogen Life Technologies), referred to as complete growth medium (GM).

### Flow cytometry

Fresh PBMC were stained using the following fluorochrome labeled mouse or rat anti-human antibodies: FITC anti-TNF-receptor II (R&D), PE anti-FOXP3 (clone PCH101), Pacific Blue anti CD3, APC-Alexa Fluor 750 anti-CD127 (all Ebioscience), APC anti-CD25, PerCP anti CD4 (BD systems), ECD anti-CD45R0 (Beckman-Coulter), and appropriate isotype controls.

IL-10 and IFN-γ production by PBMC from 30 children with acute *P. falciparum* (D0) was assessed after 5 hours stimulation in GM containing PMA (50 ng/ml) and Ionomycin (1000 ng/ml) or GM alone.

Cells were stained with FITC anti-IFN-γ, PE anti-FOXP3, PE-Cy7 anti-CD25, APC-AF750 anti-CD8, Pacific Blue anti-CD3 (all Ebioscience), PerCP anti-CD4, APC anti-IL-10 (both BD), and ECD anti-CD45R0 (Beckman-Coulter). To ascertain specificity of the intracellular cytokine staining, aliquots of some samples were incubated with saturating amounts of purified non-labelled antibody of the same clone prior to staining with the fluorochrome labeled ICS antibody. The FOXP3 staining buffer set (Ebioscience) was used following the manufacturer's protocol. Samples were acquired on a 3 laser/9 channel CyAn™ ADP flowcytometer using Summit 4.3 software (Dako). Analysis was performed using FlowJo (Tree Star Inc.). All flowcytometric analysis was performed at the MRC laboratories, The Gambia on freshly isolated cells.

### Multiplex analysis of plasma cytokine concentration

Plasma concentrations of IFN-γ, TNF-α and IL-10 were determined for each subject and time point on the Bio-Plex® 200 system, using X-Plex™ assays (both Bio-Rad Laboratories), according to the manufacturer's instructions. Data were analysed using the Bio-Plex® Manager software. The detection limit was defined as the concentration corresponding to a fluorescence value above the mean background fluorescence in control wells plus 3 SD, being 8.76 pg/ml for IFN-γ, 5 pg/ml for TNF-α and 0.57 pg/ml for IL-10. Values below this threshold were set to these levels.

### *Plasmodium falciparum* culture and schizont antigens

*P. falciparum* parasites (3D7 strain) were cultured in vitro as described [73] and were routinely shown to be mycoplasma free by PCR (Bio Whittaker). Schizont-infected erythrocytes were harvested from synchronized cultures by centrifugation through a Percoll gradient (Sigma-Aldrich). PfSE was prepared by two rapid freeze-thaw cycles in liquid nitrogen and a 37°C water bath. Extracts of uninfected erythrocytes (uRBC) were prepared in the same way.

### Proliferation assay

PBMC from 10 severe and 10 uncomplicated malaria cases collected on day 0 were depleted of CD25^hi^ cells or mock depleted using magnetic beads (Dynal Biotech, UK), at a bead to PBMC ratio of 7∶1, and cell proliferation was determined by [^3^H]-thymidine (Amersham, UK) incorporation after 6 days in culture with PfSE, uRBC (RBC∶PBMC ratio equivalent to 2∶1), GM, or 2 days culture with PMA (10 ng/ml)+ Ionomycin (100 ng/ml), as described [27].

### Cultured ELISpot

Cultured ELISPOTs were performed to assess malaria specific IFN-γ memory responses, adapting an established method [74]. Up to 1 million PBMCs collected on day 28 were cultured in 24 well plates for 6 days in 1 ml GM and stimulated with either PfSE, uRBC (RBC∶PBMC ratio equivalent to 2∶1), or GM respectively. At day 3, half the medium was exchanged and rIL-2 (final concentration 20 IU/well) was added. On day 6 cells were harvested, washed three times, and 1.5×10^5^ cells seeded into duplicate wells onto Millipore MAIP S45 plates and restimulated overnight with PfSE, uRBC (concentrations as above), GM or PHA-L (5 µg/ml). IFN-γ ELISpot was performed using MabTech antibodies according to the manufacturer's instructions. Spot forming cell numbers were counted using an ELISPOT plate reader (AutoImmuneDiagnostica, Vers. 3.2). Results are expressed as spot forming units (SFU) per million PBMC after subtraction of individual background values (GM for PHA-L, uRBC for PfSE) being deducted. Assays were discounted if the positive control (PHA-L) was <50 SFU, or the negative control was >30 SFU.

### RT-PCR

For quantitative reverse transcription-polymerase chain reaction (RT-PCR), total RNA was extracted from PAX tubes following the manufacturer's instructions and reverse transcribed into cDNA using TaqMan® reagents for reverse transcription (Applied Biosystems), following the manufacturer's protocol. Gene expression profiles for *FOXP3*, *IL-10*, *SOCS-2* and *IFN-γ* were measured by RT-PCR on a DNA Engine Opticon® (MJ Research) with QuantiTect SYBR Green PCR kits (Qiagen Ltd) using primers (all Sigma Genosys) previously described: *IFN-γ*, *IL-10*; *FOXP3* designed by [75], and *SOCS-2* designed by [76].

*T-BET* and *GATA-3* gene expression was determined using the TaqMan® Probe kit using the primers (all Metabion) designed by [77]. 18S rRNA, amplified using a commercially available kit (rRNA primers and VIC labeled probe, Applied Biosystems), was used as an internal control. Data were analysed using Opticon Monitor 3™ analysis software (BioRad) and are expressed as the ratio of the transcript number of the gene of interest over the endogenous control, 18S rRNA.

### Parasite genotyping

Genomic DNA from each parasite isolate was genotyped by sequencing the highly polymorphic block 2 region of the *msp1* gene to assess the number of clones infecting each patient [78].

### Statistical analysis

Analysis was performed using linear regression, with a random effect to allow for the within subject measurements over time, where the response variables were log transformed to improve the normality and constant variance assumptions. Significance (measured at the 5% level) tests for the effects of malaria group (uncomplicated, S_A_ or S_B_), time (day 0 and day 28) and their interaction were adjusted for the possible confounding effects of age, sex, duration of prior symptoms and numbers of clones causing the infection. Where there was no significant malaria group and time interaction, p-values for the overall comparison of day 0 vs. day 28 are given. Within day 0, comparisons of severe vs. uncomplicated and the two groups of severely ill patients (S_A_ vs S_B_) were adjusted for any malaria group and time interactions. To allow for the multiplicity of tests resulting from multiple responses and multiple comparisons within a response performed in the model, a false discovery rate (FDR) of 5% was assumed. Using the Benjamini and Hochberg approach [43] only tests with a p-value below 0.012 have an FDR of ≤5%. Due to the large number of tests family-wise error rate correction methods were too conservative. Analyses were performed using Stata version 9 and Matlab version R2008a.

## Supporting Information

Figure S1 mRNA levels for IFN-γ, IL-10 and Th-1 as well as Th-2 lineage transcription factors in severe and mild disease.
(1.99 MB TIF)
