# Supplementary figures and images for: Distinct Roles for FOXP3+ and FOXP3− CD4+ T Cells in Regulating Cellular Immunity to Uncomplicated and Severe Plasmodium falciparum Malaria
Source: PLoS Pathog. 2009 Apr 3;5(4):e1000364. doi: 10.1371/journal.ppat.1000364 (PMC2658808; doi:10.1371/journal.ppat.1000364)

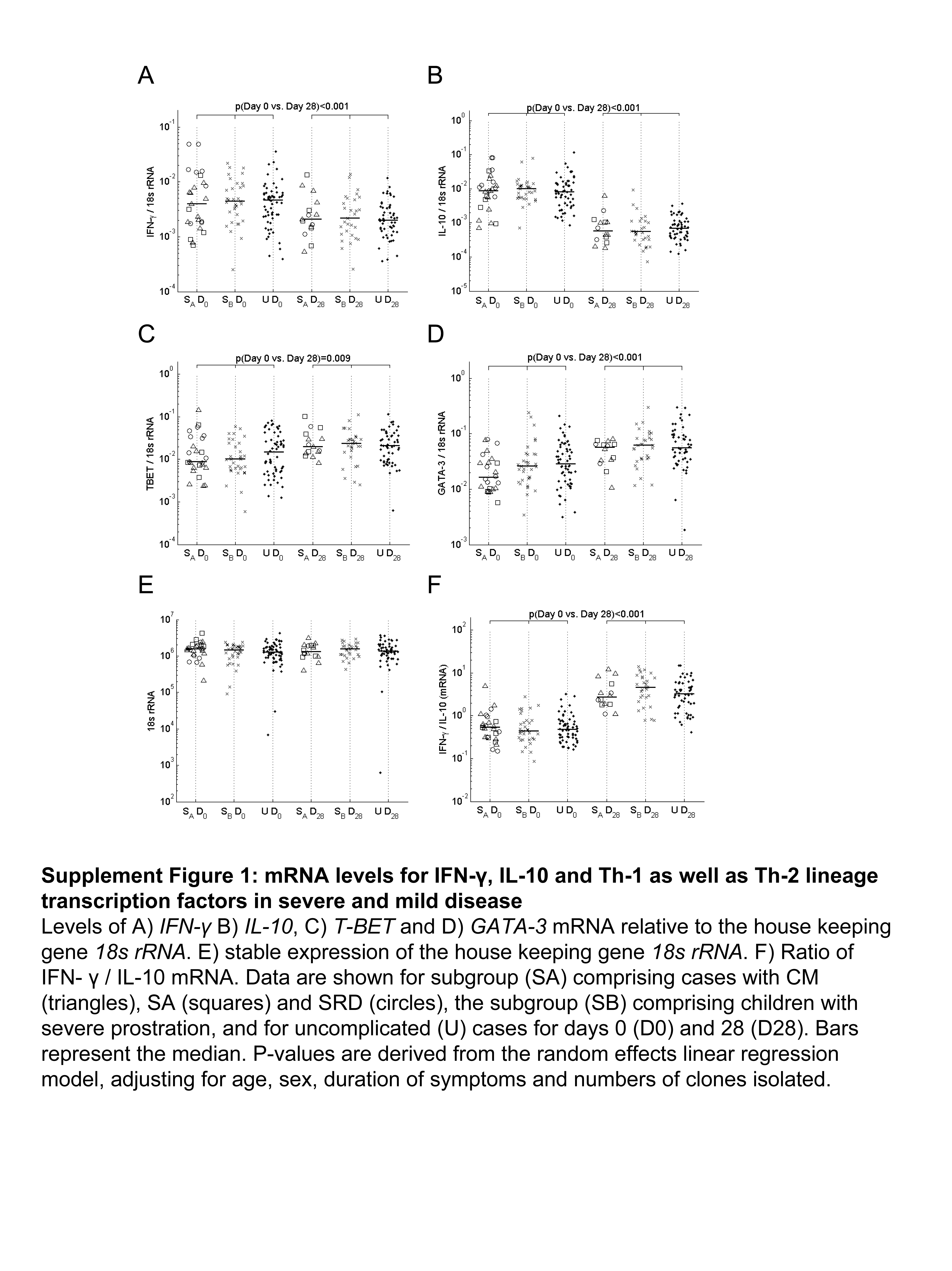

Supplement: Figure S1 — mRNA levels for IFN-γ, IL-10 and Th-1 as well as Th-2 lineage transcription factors in severe and mild disease. (1.99 MB TIF) [file ppat.1000364.s001.tif]
